# Chromatic Quantum Contextuality

**DOI:** 10.3390/e27040387

**Published:** 2025-04-05

**Authors:** Karl Svozil

**Affiliations:** Institute for Theoretical Physics, TU Wien, Wiedner Hauptstrasse 8-10/136, 1040 Vienna, Austria; karl.svozil@tuwien.ac.at

**Keywords:** contextuality, logic, hypergraph, chromatic number

## Abstract

Chromatic quantum contextuality is a criterion of quantum nonclassicality based on (hyper)graph coloring constraints. If a quantum hypergraph requires more colors than the number of outcomes per maximal observable (context), it lacks a classical realization with *n*-uniform outcomes per context. Consequently, it cannot represent a “completable” noncontextual set of coexisting *n*-ary outcomes per maximal observable. This result serves as a chromatic analogue of the Kochen-Specker theorem. We present an explicit example of a four-colorable quantum logic in dimension three. Furthermore, chromatic contextuality suggests a novel restriction on classical truth values, thereby excluding two-valued measures that cannot be extended to *n*-ary colorings. Using this framework, we establish new bounds for the house, pentagon, and pentagram hypergraphs, refining previous constraints.

## 1. Contexts as Maximal Observables

In operator-valued arguments, a context can be seen as being capable of potentially encoding and utilizing the *maximal knowledge* of a system—all that can be conceivably extracted from a single ‘maximal’ measurement [1].

A context can, through the spectral theorem, be identified using an orthonormal basis with the elements $|e_{i}\rangle$ or alternatively the associatedmutually orthogonal self-adjoint projection operators $|e_{i}\rangle \langle e_{i}|$. These can be bundled together through a non-degenerate spectral sum (decomposition) as $\sum_{i}a_{i}|e_{i}\rangle \langle e_{i}|$ with real-valued $a_{i}$ which are mutually distinct to form the self-adjoint *maximal* operator introduced by von Neumann ([2], Satz 8, p. 221f); a good description is given by Halmos ([3], § 84, p. 171f).

A maximal (and thus non-degenerate) operator, or its corresponding context, represents the totality of what can ideally be measured—no more, no less. In this case, the measurement *resolution* is at its finest, corresponding to individual basis elements $|e_{i}\rangle$. Equivalently, it pertains to the (mutually orthogonal) one-dimensional subspaces spanned by these basis elements and the respective property of “being in the state $|e_{i}\rangle$”.

In contrast, a two-valued measure resolves a single one-dimensional subspace spanned by $|e_{i}\rangle$, assigning it the value 1, while the rest, an (n−1)-dimensional subspace in an *n*-dimensional Hilbert space, is assigned the value 0. Any orthonormal basis element of this (n−1)-dimensional subspace is assigned the value 0.

Therefore, *coloring* with mutually different numbers, parameters, outcomes, or colors $a_{i}$ yields not only a finer resolution than two-valued states can offer but also corresponds to the optimal experimental extraction of the data from a state by a maximal operator. In physics, we need to insist on (at least in principle) the maximal conceivable resolution—all that can, at least in principle, be measured.

## 2. The Connection to the Chromatic Number of Hypergraphs

Hypergraphs [4] are extensively utilized in quantum logics to model propositions and the contexts (Boolean subalgebras) to which they belong [5,6,7,8]. In this framework, each Boolean subalgebra—also known as a block, maximal operator, orthonormal basis, or context—is represented by a hyperedge in the hypergraph. These hyperedges are visually depicted as smooth lines connecting the vertices that correspond to the propositions within that subalgebra. The structure of the hypergraph captures the orthogonality relations among propositions, where propositions within the same hyperedge are pairwise orthogonal [9,10]. For a recent detailed exposition of these concepts, including precise definitions and illustrative examples, the reader is referred to Ref. [11].

In the context of hypergraph coloring, each maximal observable corresponds to a hyperedge, and the vertices represent the possible outcomes or states associated with that observable. The *exclusivity* requirement—that no hyperedge can have two or more of its vertices colored the same—reflects the quantum mechanical constraint that a maximal observable cannot yield two or more outcomes for any of its possible eigenstates.

A further *completeness* requirement states that all colors must occur in each hyperedge, meaning that no hyperedge lacks a color necessary for coloring the entire hypergraph. Thus, the chromatic number of the hypergraph provides a measure of the minimal number of distinct outcomes needed to satisfy these constraints.

A coloring of a hypergraph is said to be *admissible* if it is both exclusive and complete. If no admissible coloring exists—that is, if the chromatic number exceeds the number of vertices per hyperedge—this indicates that no conceivable and possible coloring (relative to admissibility, that is, the exclusivity and completeness rules) exists. This ‘excess of required outcomes’, in turn, highlights the nonclassical nature of the respective collection of quantum observables corresponding to the hypergraph (with vector vertex labels).

In what follows, we shall only consider *n-uniform* hypergraphs with an equal number *n* of vertices per edge. As argued earlier, every edge of such a hypergraph can be identified with a context and a maximal observable. We shall consider colorings of such hypergraphs as the assignment of colors to its vertices such that no hyperedge has vertices with the same color: every hyperedge contains vertices in *n* different colors.

The chromatic number *k* of a hypergraph is the *minimal* number of colors required to achieve a coloring that satisfies exclusivity. Note that this does not necessarily mean that the coloring satisfies completeness. We note in passing that unlike the terminology used here, a proper coloring of a hypergraph often refers to the assignment of colors to its vertices such that each hyperedge contains at least two vertices of different colors.

Moreover, a *k*-uniform proper coloring of a hypergraph is the assignment of colors to its vertices such that all colors are assigned to an equal number of vertices [12]. This implies that the assignment of colors partitions the set of vertices into disjoint subsets of an equal size.

If k=n, we obtain a ‘canonical’ *k*-uniform proper coloring of *n*-uniform hypergraphs, which are particularly important for physics: Any such coloring can be reduced into a two-valued state through *aggregation*: assigning a single color the value 1 while mapping all other k−1 colors to 0 [13]. However, the inverse is not possible: As will be discussed later, the mere existence of two-valued states does not guarantee a chromatic number *n* ([11], Appendix B, p. 032104-16f).

Any admissible coloring—respecting both exclusivity and completeness—corresponds to a value assignment that maintains the maximal resolution within each context. In the hypergraph coloring framework, each maximal observable is represented by a hyperedge, with the vertices corresponding to its possible outcomes or eigenstates. The constraint that no hyperedge can contain two or more identically colored vertices reflects the quantum mechanical principle that a maximal observable cannot yield identical outcomes for distinct eigenstates. Additionally, each hyperedge must include all of the colors needed to properly color the hypergraph. Since the chromatic number of the hypergraph quantifies the exact number of distinct outcomes required to meet these constraints, any deviation exceeding the uniform number of vertices per hyperedge indicates the nonclassical nature of quantum systems.

If we can identify a quantum-representable hypergraph—one that permits a faithful orthogonal representation [9,10,14]—that is uniform with *n* vertices per hyperedge and has a chromatic number exceeding *n*, then we can demonstrate that this configuration does not correspond to a physically realizable (classical counterfactual) measurement setup with noncontextual, coexisting uniform outcomes. We may perceive this as a form of *chromatic contextuality*.

Chromatic contextuality differs from the theorems of Kochen–Specker, Greenberger–Horne–Zeilinger [15], or Hardy’s paradox [16], which can all be expressed as arguments involving two-valued states, although some of them can be written in terms of operator values. The mere existence of a two-valued state—amounting to, within a given context, assigning a unique value (say, ‘1’) to one outcome and a different, single value (say, ‘0’) to all other outcomes—is, by itself, insufficient to prove that mutually distinct outcomes—associated with that context’s maximal observable—pre-exist; in particular, when demanding uniform consistency across all possible intertwining contexts (thereby necessarily involving counterfactual nondegenerate outcomes also for unchosen measurements). This is true even if the set of two-valued states is separable—meaning that at least one two-valued state exists that separates every pair of vertices—because such configurations may not allow for the potential acquisition (through counterfactual experiments) of the maximal knowledge (per context).

Clearly, in Kochen–Specker cases, there is no two-valued state and thus no coloring. However, we might hope to find ‘smaller hypergraphs’ (with a ‘small’ number of edges or vertices) that have no coloring with *n* colors but still have (even a separating set of) two-valued states conforming to the demarcated Theorem 0 of Kochen and Specker [17].

## 3. Previous Results

A set representable hypergraph exists that does not allow for a coloring whose required minimal number of colors exceeds the number of vertices per edge: the corresponding graph $G_{32}$ was initially discussed by Greechie ([5], Figure 6, p. 121) (see also Refs. [18,19,20,21]). $G_{32}$ is a three-uniform hypergraph with 15 bi-intertwined vertices in 10 contexts. It supports a separating set of two-valued states. Its chromatic number is 4 ([11], Appendix B, p. 032104-16f).

## 4. The Chromatic Number of the Yo-Oh Hypergraph

**Figure 1:**
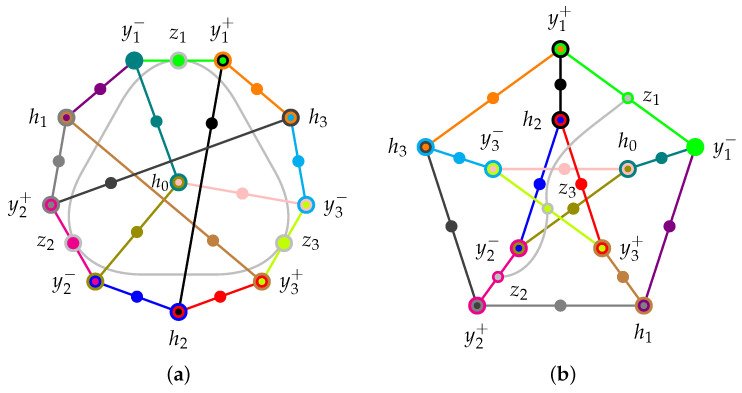
Redrawing ([23], Chapter 12, p. 92) of two equivalent representations (**a**) and (**b**) of a Petersen graph-like (with one additional context connecting $z_{1}$, $z_{2}$, and $z_{3}$) hypergraph of the logic considered by Yu and Oh ([22], Figure 2). The set of two-valued states enforces that at most one of the four atoms $h_{0}$, $h_{1}$, $h_{2}$, and $h_{3}$ is 1. The logic has a (quantum) realization in $R^{3}$ consisting of the 25 projections associated with the one-dimensional subspaces spanned by the 13 vectors from the origin ${\left(0,0,0\right)}^{⊺}$ to $z_{1}={\left(1,0,0\right)}^{⊺}$, $z_{2}={\left(0,1,0\right)}^{⊺}$, $z_{3}={\left(0,0,1\right)}^{⊺}$, $y_{1}^{-}={\left(0,1,-1\right)}^{⊺}$, $y_{2}^{-}={\left(1,0,-1\right)}^{⊺}$, $y_{3}^{-}={\left(1,-1,0\right)}^{⊺}$, $y_{1}^{+}={\left(0,1,1\right)}^{⊺}$, $y_{2}^{+}={\left(1,0,1\right)}^{⊺}$, $y_{3}^{+}={\left(1,1,0\right)}^{⊺}$, $h_{0}={\left(1,1,1\right)}^{⊺}$, $h_{1}={\left(-1,1,1\right)}^{⊺}$, $h_{2}={\left(1,-1,1\right)}^{⊺}$, and $h_{3}={\left(1,1,-1\right)}^{⊺}$, respectively [22].

In a proof through contradiction, suppose that the Yo-Oh configuration [22] of quantum observables (propositions), as depicted using a three-uniform hypergraph in a previous publication ([23], Chapter 12, p. 92) and is redrawn in Figure 1, can be colored with three colors: red, green, and blue.

Without the loss of generality, we can assume $h_{0}$ is red. Then, $y_{1}^{-}$, $y_{2}^{-}$, and $y_{3}^{-}$ must be either green or blue.

Assuming that all are colored green leads to a contradiction, as the context $\left\{z_{1},z_{2},z_{3}\right\}$ would then lack the color green.

**Figure 2:**
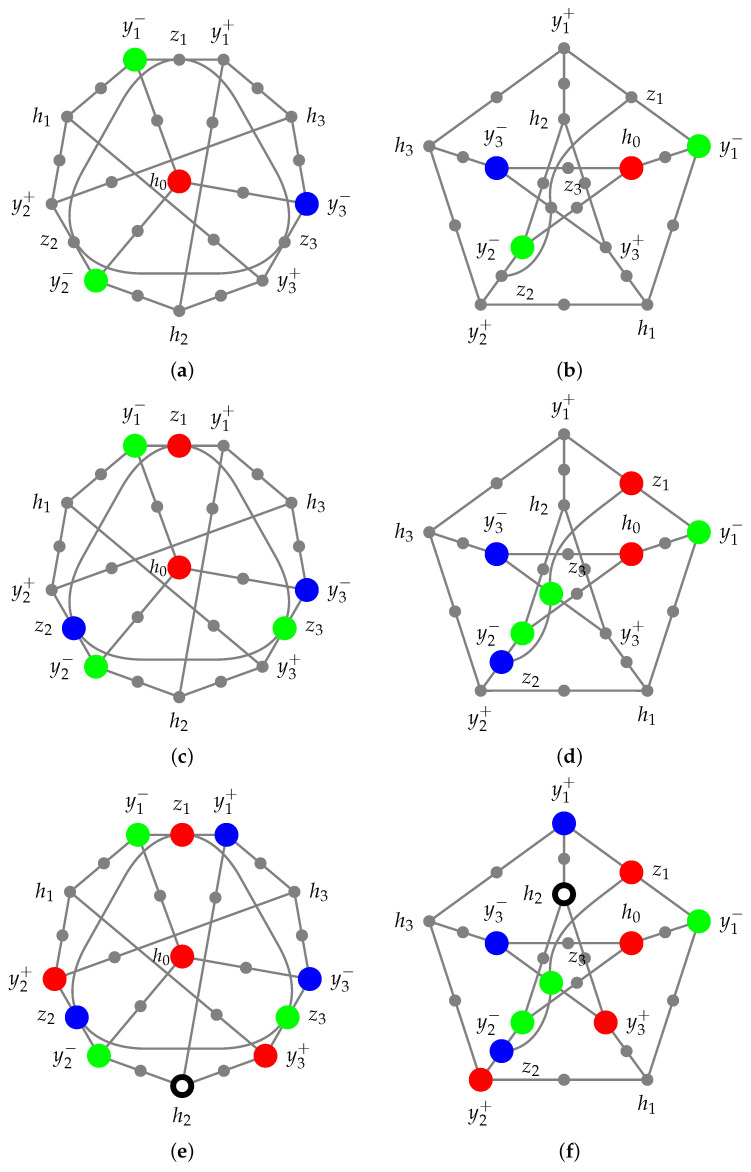
Case 1 of the proof that the Yu-Oh hypergraph depicted in Figure 1 cannot be (noncontextually) colored with three colors: its chromatic number is four.

**Figure 3:**
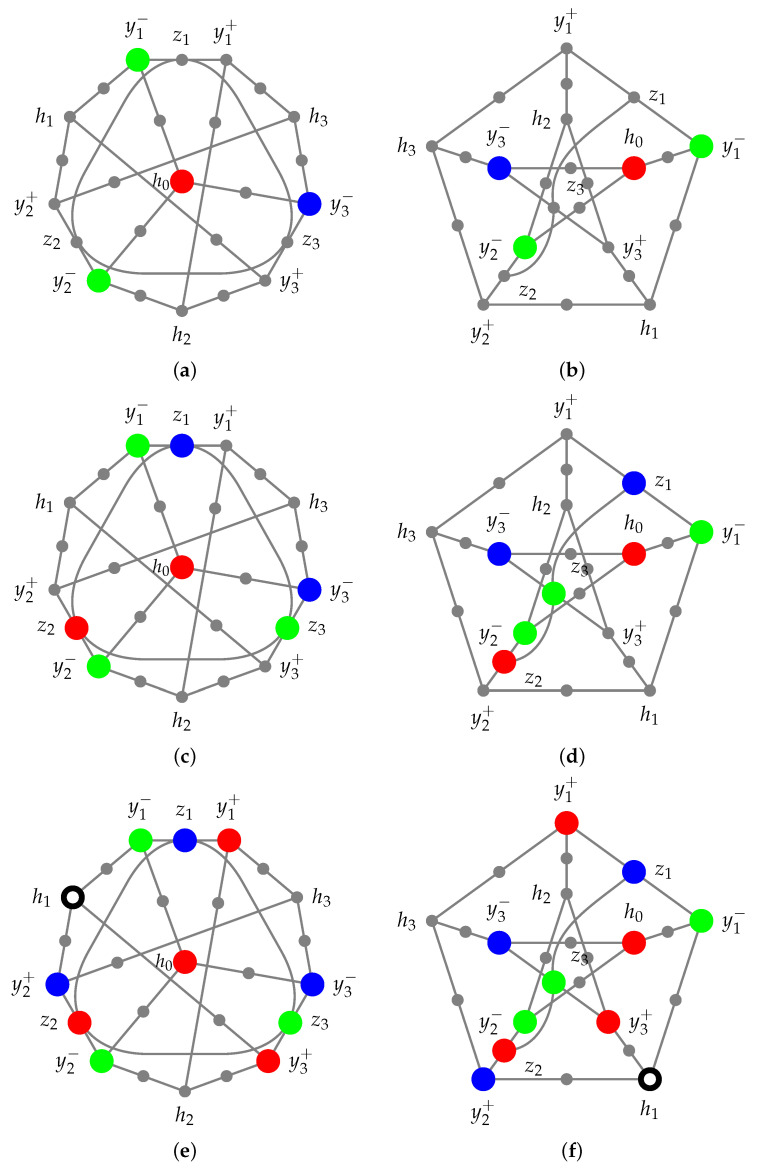
Case 2 of the proof that the Yu-Oh hypergraph depicted in Figure 1 cannot be (noncontextually) colored with three colors: its chromatic number is four.

Therefore, at least one of $y_{1}^{-}$, $y_{2}^{-}$, and $y_{3}^{-}$ must be colored differently. According to symmetry, without a loss of generality, let $y_{1}^{-}$ and $y_{2}^{-}$ be green and $y_{3}^{-}$ be blue, as depicted in Figure 2 and Figure 3, (a) and (b), respectively. Then, $z_{1}$ as well as $z_{2}$ cannot be green, and $z_{3}$ cannot be blue.

### 4.1. Case 1

Suppose that $z_{1}$ is red. Then, $z_{2}$ must be blue, and $z_{3}$ must be green, as depicted in Figure 2c,d. We can now assign colors to the three contexts $\left\{y_{1}^{+},y_{1}^{-},z_{1}\right\}$, $\left\{y_{2}^{+},y_{2}^{-},z_{2}\right\}$, and $\left\{y_{3}^{+},y_{3}^{-},z_{3}\right\}$ by assigning blue to $y_{1}^{+}$, red to $y_{2}^{+}$, and red to $y_{3}^{+}$, respectively, as depicted in Figure 2e,f.

Consequently, $h_{2}$ cannot be red because $y_{3}^{+}$ is red, nor can it be green, as $y_{2}^{-}$ is green, nor can it be blue, as $y_{1}^{+}$ is blue, and all $y_{3}^{+}$, $y_{2}^{-}$, and $y_{1}^{+}$ are adjacent to $h_{2}$.

### 4.2. Case 2

Suppose that $z_{1}$ is blue. Then, $z_{2}$ must be red, and $z_{3}$ must be green, as depicted in Figure 3c,d.

Consequently, $y_{1}^{+}$ must be red, $y_{2}^{+}$ must be blue, and $y_{3}^{+}$ must be red. Additionally, $h_{3}$ must be green, and $h_{2}$ must be blue. As before, we can now assign colors to the three contexts $\left\{y_{1}^{+},y_{1}^{-},z_{1}\right\}$, $\left\{y_{2}^{+},y_{2}^{-},z_{2}\right\}$, and $\left\{y_{3}^{+},y_{3}^{-},z_{3}\right\}$ by assigning red to $y_{1}^{+}$ and $y_{3}^{+}$ and blue to $y_{2}^{+}$, respectively, as depicted in Figure 3e,f.

Now, $h_{1}$ cannot be red because $y_{3}^{+}$ is red, nor can it be green, as $y_{1}^{-}$ is green, nor can it be blue, as $y_{2}^{+}$ is blue, and all $y_{3}^{+}$, $y_{1}^{-}$, and $y_{2}^{+}$ are adjacent to $h_{1}$.

It is not difficult to work out a coloring for the Yu-Oh hypergraph with four colors. Therefore, its chromatic number is four. In passing, we note that it has a separating set of 24 two-valued measures.

## 5. Summary

Chromatic contextuality, characterized by the impossibility of admissibly coloring an *n*-uniform hypergraph with *n* colors per hyperedge, presents a compelling case against the existence of classical (noncontextual) hidden parameters. In this way, chromatic contextuality resembles Kochen–Specker contextuality, which is defined by the absence of uniform two-valued states [17,24], or admissible states [25]. Chromatic contextuality can be viewed as an extreme form of operator-valued argument given that the number of values involved is equivalent to the number of vertices in each context edge of the hypergraph.

Chromatic contextuality, in contrast to Kochen–Specker contextuality, constitutes a distinct criterion for nonclassicality: the respective hypergraphs and the collection of quantum observables they represent may still support two-valued states and even permit (though not necessarily imply) classical embeddability through a separating set of two-valued states. This is exemplified by Greechie’s $G_{32}$ hypergraph, which admits a set representation in terms of a partition logic ([11], Appendix B, p. 032104-16f).

Moreover, any *n*-coloring can be directly converted into a two-valued state—indeed, into *n* two-valued states—through aggregation, that is, by reducing or folding the number of *n* colors into two. This can be carried out by identifying a single color with the value 1 and all remaining colors with 0. In this way, the set of observables encoding the hypergraph is equi-partitioned.

Alternatively, an *n*-coloring can be used by identifying more than one color with a non-zero value [26], for example, identifying two colors with the value 1/2. A single coloring thus defines a canonical set of *n* two-valued states covering the entire hypergraph ([11], Appendix A).

However, the converse is not true: the existence of even separating sets of two-valued states does not imply the existence of a coloring, as demonstrated by the aforementioned example of $G_{32}$.

It is quite remarkable that not all such colorings can be derived from the nonexclusive hypergraph coloring scheme discussed above. In particular, the exotic two-times-1/2 coloring of a pentagon (or a house or a pentagram) on intertwining context hyperedges, as exposed by Greechie ([27], Figure 5, p. 186) and Wright ([28], $\omega_{0}$, p. 268), cannot be obtained through identifying colors. The reason for this is a parity argument: For an odd number of hyperedges, such as five, a coloring with the same color assigned to all (odd-numbered) intertwining vertices cannot exist.

By the same parity reasoning, one of the eleven two-valued states of the pentagon, in which all of the values are assigned to the nonintertwining ‘middle’ vertices, does not originate from the aggregation from a hypergraph coloring. Suppose one of the colors, say red, is always centered in the middle; this would imply that the other two colors, say green and blue, must alternate at the five vertices with intertwining contexts. However, for an odd number of such intertwining vertices, this leads to a disallowed configuration; more explicitly, to green–red–blue–red–green–red–blue–red–*green*–*red*–*green* (from the cycle). If this two-valued state is eliminated, the Hull computation still yields Klyachko’s pentagram inequality ([29], Equation (5)), along with an additional upper bound: $1\geq A_{13}+A_{35}+A_{57}+A_{79}+A_{91}\geq -3$. This explicit house–pentagon–pentagram example also demonstrates that for hyperedges with more than two vertices, not all two-valued states can necessarily be derived through aggregation—the irreversible mapping of one color to the value 1 and all other colors to 0. The Bub and Stairs inequality [30] remains unaffected, as they do not use the ‘color-forbidden middle-center’ two-valued state. This topic is too broad to be fully addressed within the scope of this paper.

For physical reasons, we suggest that every two-valued state should ultimately originate from the ‘perfect’, that is, maximal, measurements corresponding to the colorings. Therefore, only two-valued states that are derived through aggregation should be considered when deriving, for instance, Boole–Bell-type inequalities by solving the Hull problem for the respective correlation polytope [31,32]. Two-valued states that cannot be represented through the aggregation of colorings are unphysical in the sense that no (classical) maximal observables or contexts exist that could justify their inclusion.

A chromatic analogue of True-Implies-False (TIF) and True-Implies-True (TIT) gadgets [33], which exhibit a distinct form of contextuality, such as Hardy-type [16], has not yet been explored in the context of hypergraph colorings. Additionally, the concept of chromatic separability, similar to Kochen–Specker’s demarcation criterion of (non)separability with respect to two-valued states, remains to be discussed. For instance, does nonseparability through two-valued states imply chromatic nonseparability? We note that Kochen and Specker’s $\Gamma_{3}$ [17] is also color-nonseparable. Is aggregation sufficient for the inheritance of such properties? These topics require future investigation.

## Data Availability

No new data were created or analyzed in this study. Data sharing is not applicable to this article.
